# Manipulation of Photoelectrochemical Water Splitting by Controlling Direction of Carrier Movement Using InGaN/GaN Hetero-Structure Nanowires

**DOI:** 10.3390/nano13020358

**Published:** 2023-01-16

**Authors:** Siyun Noh, Jaehyeok Shin, Yeon-Tae Yu, Mee-Yi Ryu, Jin Soo Kim

**Affiliations:** 1 Department of Electronic and Information Materials Engineering, Division of Advanced Materials Engineering, Research Center of Advanced Materials Development, Jeonbuk National University, Jeonju 54896, Republic of Korea; 2 Department of Physics, Kangwon National University, Chuncheon 24341, Republic of Korea

**Keywords:** photoelectrochemical water splitting, InGaN/GaN hetero-structure nanowires, photocathode, Si, interface properties

## Abstract

We report the improvement in photoelectrochemical water splitting (PEC-WS) by controlling migration kinetics of photo-generated carriers using InGaN/GaN hetero-structure nanowires (HSNWs) as a photocathode (PC) material. The InGaN/GaN HSNWs were formed by first growing GaN nanowires (NWs) on an Si substrate and then forming InGaN NWs thereon. The InGaN/GaN HSNWs can cause the accumulation of photo-generated carriers in InGaN due to the potential barrier formed at the hetero-interface between InGaN and GaN, to increase directional migration towards electrolyte rather than the Si substrate, and consequently to contribute more to the PEC-WS reaction with electrolyte. The PEC-WS using the InGaN/GaN-HSNW PC shows the current density of 12.6 mA/cm^2^ at −1 V versus reversible hydrogen electrode (RHE) and applied-bias photon-to-current conversion efficiency of 3.3% at −0.9 V versus RHE. The high-performance PEC-WS using the InGaN/GaN HSNWs can be explained by the increase in the reaction probability of carriers at the interface between InGaN NWs and electrolyte, which was analyzed by electrical resistance and capacitance values defined therein.

## 1. Introduction

The photoelectrochemical water splitting (PEC-WS) is considered as an effective candidate for efficient production of hydrogen without emitting greenhouse gases [1]. The PEC-WS is typically conducted in a harsh electrolyte solution under solar irradiation. For the efficient production of hydrogen gas, photo-generated carriers inside a photocathode (PC) should be extracted as much as possible to reduce protons at the interface with electrolyte [2]. Therefore, to implement PEC-WS more efficiently, new PC materials and structures, having superior light absorption for the generation of a large number of carriers and high chemical stability, should be suggested and developed. The III-nitride material system is one of the promising candidates for the PC medium to get high-performance PEC-WS, because its band-edge position can cover the proton-reduction and water-oxidation potentials, indicating possibility for the PEC-WS reaction without external bias [3]. For example, the energy-band gap of In*_x_*Ga_1−*x*_N (hereafter InGaN) can be modulated from 0.7 to 3.4 eV by controlling indium (In) content [4], resulting that the number of carriers generated by light absorption can be effectively controlled. Moreover, InGaN has high stability to corrosion in harsh acidic or alkaline electrolytes [5]. If the III-nitride material is fabricated into a nanowire (NW) structure, the PEC-WS reaction can be improved because it has a relatively high surface-to-volume ratio compared to its bulk structure or thin film [6]. Recently, the III-nitride NWs are largely grown on a Si substrate [7,8]. However, it is very challenging work to grow high-crystalline III-nitride NWs mainly due to the significant difference in lattice constant and thermal expansion coefficient, etc. [9]. That is, the crystallinity of the III-nitride NWs, including InGaN NWs, is very poor due to the generation of a large number of defects (stacking faults) formed during the growth process [10]. In addition, upward energy-band bending occurs at the surface of the III-nitride NWs [11], leading to limiting the transfer rate of photo-generated electrons to the electrolyte and hydrogen evolution reaction (HER). As a result, there have been only few reports on the PEC-WS using III-nitride NWs as a PC material. To overcome the upward energy-band bending at the NW surface and to improve the PEC-WS performance, several research groups suggested p-type doping for the NWs [12,13]. However, it remains a challenging task to increase the p-type doping level with more than 10^18^/cm^3^ for the NWs [14]. As an alternative to suppress the energy-band bending, the NWs were coated with various metal catalysts [15,16,17,18]. For example, Vanka et al. demonstrated the passivation of the InGaN NWs with Pt and Au catalysts to enhance the charge-transfer rate [15]. However, the light absorption of the PCs fabricated with InGaN NWs is intrinsically limited because the use of metal catalysts reduces the amount of light reaching the NWs. In addition, there are some serious problems such as backward reaction and dissolution of metal catalysts during the PEC-WS reaction, which significantly impede the HER and degrade time-dependent performance [16,17,18]. Based on this consideration, it would be better to use high-crystalline III-nitride NWs alone without using any metal catalyst to improve PEC-WS performance.

In this paper, we suggest a new way to improve PEC-WS using undoped InGaN/GaN hetero-structure NWs (HSNWs) as a PC material. Any metal catalyst is not used for the fabrication of the InGaN/GaN-HSNW PCs. The InGaN/GaN HSNWs can control the migration kinetics of photo-generated carriers due to the potential barrier formed at the hetero-interface between InGaN and GaN, and consequently, increase the possibility for the reaction between electrons and electrolyte. For the measurement of PEC-WS, the current density (*J*)–voltage (*V*) characteristic curves of the InGaN/GaN-HSNW PCs were recorded with a three-electrode configuration in 0.5-M H_2_SO_4_ electrolyte. The carrier-transfer rate was investigated by approximating the interface between the InGaN/GaN HSNW PC and the electrolyte as an equivalent circuit with a resistance and a capacitance, quantitatively obtained from the Nyquist plots.

## 2. Experimental Methods

**Figure 1:**
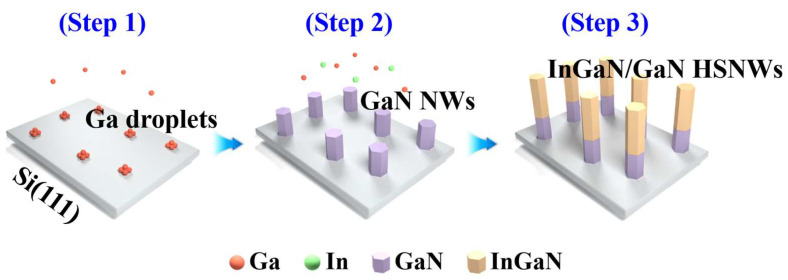
Schematic diagram for the growth of the InGaN/GaN HSNWs.

The undoped InGaN/GaN HSNWs were formed on a 275-μm-thick p-Si(111) substrate using a plasma-assisted molecular-beam epitaxy (PA-MBE) system. Before loading the Si substrate into the PA-MBE chamber it was cleaned in a solution of HCl:H_2_O_2_:H_2_O (1:1:6 by volume) for 10 min. After the cleaning process the oxidation layer naturally formed on the Si(111) substrate was removed by in situ annealing at a substrate temperature of 900 °C. A schematic diagram for the growth process of undoped InGaN/GaN HSNWs is shown in Figure 1. As a first step, gallium (Ga) droplets are formed only by supplying Ga flux on the Si(111) substrate (step 1). This process is the *so-called* Ga pre-deposition method and was reported in our previous studies [9,19,20,21,22]. Next, Ga flux is supplied under nitrogen (N)-plasma condition to form GaN NWs (step 2). The formation method of the NWs used in this work is quite different from the conventional techniques such as vapor–liquid–solid growth mode using metal catalysts and chemical-etching process [23,24]. After forming GaN NWs with the average length of 109 ± 5 nm, which was calculated from the NWs in the area of 1 × 1 μm^2^, as nucleation seeds, In and Ga fluxes are simultaneously supplied under N-plasma condition to form InGaN NWs (step 3). The In content of InGaN/GaN HSNWs is varied by changing In flux (4.2 × 10^−8^, 2.1 × 10^−7^, and 3.5 × 10^−7^ Torr) at a fixed Ga flux (3.5 × 10^−7^ Torr). For comparison, only GaN NWs were also formed on Si and denoted as a Ref-NW sample.

To analyze the structural properties of the NW samples, field-emission scanning-electron microscopy (FE-SEM; Hitachi), X-ray diffraction (XRD; Rigaku), and aberration-corrected transmission-electron microscopy (Cs-TEM; Jeol) measurements were used. The FE-SEM (Hitachi S-4700, installed in the Future Energy Convergence Core Center at Jeonbuk National University) measurement was performed at the accelerating voltage of 10 kV. The XRD (MAX-2500) rocking curves of the NW samples were obtained with a wavelength of Cu *K_α_* (1.54 Å). The Cs-TEM system (Jeol Jem-arm 200 F) was used to investigate the crystallinity of the NW samples. The optical properties of the NW samples were analyzed using a photoluminescence (PL) spectroscopy and an ultraviolet–visible spectrophotometer. The PL spectra were obtained with a diode-pumped solid-state laser with a wavelength of 266 nm as an excitation source. The length of a monochromater was 0.5 m, and luminescence from the NW samples was detected by a charge-coupled device. The ultraviolet-visible spectrophotometer (UV-2550; Shimadzu) was used to measure the absorbance of the NW samples, where the slit size was set to 5 nm. The baseline was first set using two Si substrates before the absorbance measurement of the NW samples. Thereafter, one of the Si substrates was replaced to the NW samples, and the absorbance of the NW samples were obtained. The obtained absorbance of the NW samples was converted to arbitrary units by normalization. The PEC-WS using Ref-NW and InGaN/GaN-HSNW photoelectrodes was performed with a three-electrode configuration in 0.5-M H_2_SO_4_ electrolyte. The PEC-WS cell is composed of a NW sample, Ag/AgCl, and Pt serving as a working electrode, a reference electrode, and a counter electrode, respectively. A xenon lamp (MAX-303; Asahi Spectra) was used as a light source. The active area of the NW samples used as a working electrode was 0.5 × 0.5 cm^2^. A potentiostat (Reference-3000; Garmy) was used to record the PEC-WS performance of the NW-based photoelectrodes at the potential ranging from −1 to 1 V versus reversible hydrogen electrode (RHE). To evaluate the carrier-transfer rate of the NW-based photoelectrodes, Nyquist plots, measured by electrochemical impedance spectroscopy, are investigated. The electrochemical impedance measurements were performed by applying −1 V versus RHE at a frequency range of 100 kHz to 0.1 Hz with an amplitude of 10 mV under illumination condition.

## 3. Results and Discussion

**Figure 2:**
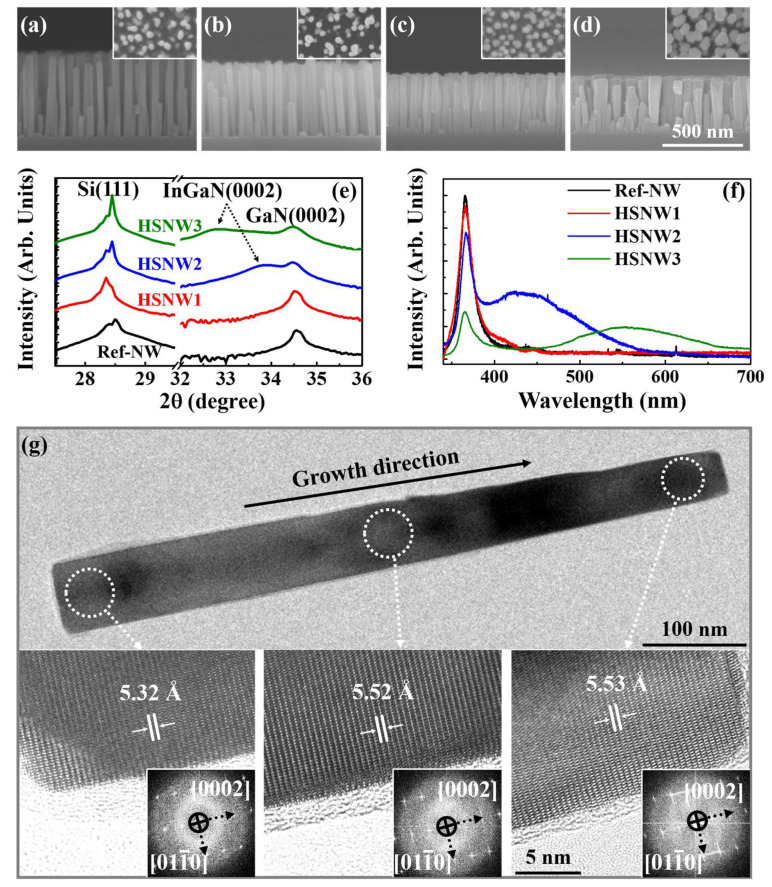
Cross-sectional FE-SEM images of the (**a**) Ref-NW, (**b**) HSNW1, (**c**) HSNW2, and (**d**) HSNW samples, where the insets are the plan-view images. (**e**) Normalized XRD rocking curves and (**f**) PL spectra of the Ref-NW, HSNW1, HSNW2, and HSNW3 samples. (**g**) Cs-TEM image (**top**), HR-TEM images (**bottom**), and the SAED patterns (**inset**) for the (**top**), (**middle**), and the (**bottom**) regions of the InGaN/GaN HSNW.

Figure 2a–d show the cross-sectional and plan-view (inset) FE-SEM images of the NW samples. Figure 2e,f shows the normalized XRD rocking curves and PL spectra of the NW samples, respectively. From the XRD rocking curves and PL spectra, the In contents of the InGaN/GaN HSNWs were measured as 6.4% (denoted as HSNW1), 13.6% (HSNW2), and 32.7% (HSNW3). In the FE-SEM images, the average heights (diameters) of the NWs for the Ref-NW, HSNW1, HSNW2, and HSNW3 samples were measured as 506.3 ± 28.2 (36.5 ± 5.4), 398.4 ± 18.4 (42.5 ± 3.1), 363.5 ± 10.5 (51.1 ± 3.4), and 326.9 ± 19.7 nm (64.8 ± 9.9 nm), respectively. The average heights (diameters) were obtained by analyzing the NWs in the area of 1 × 1 μm^2^. The spatial densities of the NWs for the Ref-NW, HSNW1, HSNW2, and HSNW3 samples were measured as 1.0 × 10^9^, 1.1 × 10^9^, 1.3 × 10^9^, and 1.4 × 10^9^/cm^2^, respectively. The above quantitative values were calculated using ‘Image J Software’ installed in the FE-SEM system. The change in the spatial density of NWs was negligible. As the In content increases, the height (diameter) of NWs was decreased (increased). These results are attributed to the decrease in the vertical growth as the In content increases, largely because the migration length of Ga adatoms becomes limited by In adatoms at the growth temperature for InGaN [25,26]. In the normalized XRD rocking curves for the Ref-NW and HSNW samples, two narrow peaks corresponding to Si(111) and GaN(0002) were observed at 28.5 and 34.6°, respectively. The peak intensity of GaN(0002) of the Ref-NW sample is relatively stronger than those of the HSNW samples due to the larger volume of the GaN NWs. Additional shoulder peaks corresponding to InGaN(0002) are observed at 33.9 and 32.8° for the HSNW2 and HSNW3 samples, respectively. This result indicates successful formation of InGaN/GaN HSNWs on the Si(111) substrate. For the HSNW1 sample, the InGaN(0002) peak is weakly observed because of very low In content. The full width at half maximum (FWHM) values of the peak corresponding to InGaN(0002) of the HSNW2 and the HSNW3 samples were measured as 0.58 and 0.61°, respectively. The FWHM values of the InGaN/GaN HSNWs in this study are much narrower compared to those (~1.5°) of previous reports [27,28]. The FWHM value of the HSNW3 sample is wider than that of the HSNW2 sample, which is related to the increase in the In segregation typically observed from InGaN with a high In content [29]. The peak intensity of InGaN(0002) for the HSNW2 sample is also stronger than that of the HSNW3 sample. In the PL spectra for the Ref-NW and HSNW samples, measured at room temperature (RT), the strong free-exciton peaks corresponding to GaN NWs were clearly observed at the wavelength of 365.8 nm. In the previous reports, the free-exciton peak was hardly observed from III-nitride NWs at RT because of the non-radiative recombination of photo-generated carriers within the NWs largely caused by defects and stacking faults [30,31].

For the HSNW1 sample, the free-exciton peak of the InGaN NWs was weakly observed at the wavelength of 397.2 nm, which is consistent with the XRD rocking curves. The InGaN free-exciton peaks for the HSNW2 and HSNW3 samples were observed at the wavelength of 425.5 and 553.9 nm, respectively. The red-shift in the peak wavelength of the HSNW3 sample from that of the HSNW2 sample is attributed to the increase in the In content. The FWHM values of the free-exciton peaks for the HSNW2 and HSNW3 samples were calculated as 127.2 and 151.2 nm, respectively. The narrower PL spectrum of the HSNW2 sample compared to the HSNW3 sample indicates its better crystal quality. This result is also in good agreement with the XRD data. Considering these results, we successfully formed high-crystalline InGaN/GaN HSNWs on the Si(111) substrate. Figure 2g shows the Cs-TEM image (top) of an InGaN/GaN HSNW for the HSNW2 sample. High-resolution TEM (HR-TEM) images (bottom) and selective-area electron-diffraction (SAED) patterns (inset) measured from the bottom (GaN), middle (InGaN), and top (InGaN) regions of the HSNW are also shown in Figure 2g. Defects and stacking faults, easily observed from III–V semiconductor NWs formed on Si [32], were hardly observed from the TEM images. The lattice spacings were measured as 5.32, 5.52, and 5.53 Å for the bottom, middle, and top regions of the InGaN/GaN HSNW, respectively, clearly indicating the formation of the InGaN/GaN HSNW. Once again, we highlight the successful formation of high-crystalline InGaN/GaN HSNWs on the Si(111) substrate using Ga pre-deposition method.

**Figure 3:**
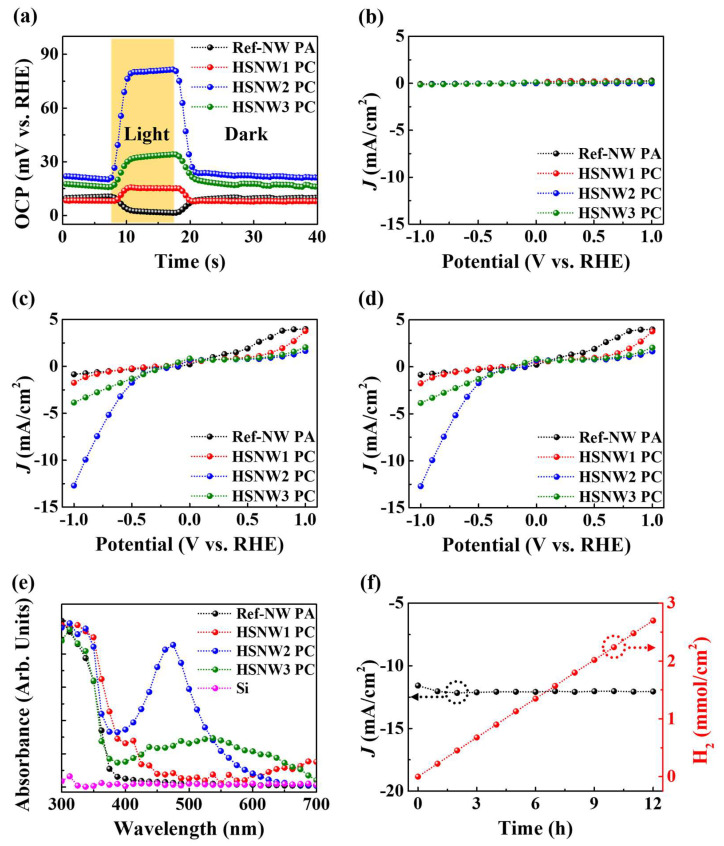
(**a**) Time-dependence of the OCP curves of the Ref-NW PA and HSNW1, HSNW2, and HSNW3 PCs in 0.5-M H_2_SO_4_ electrolyte. *J*–*V* characteristic curves of the Ref-NW PA and HSNW1, HSNW2, and HSNW3 PCs under (**b**) dark and (**c**) illumination condition. (**d**) ABPEs and (**e**) absorbance of the Ref-NW PA and HSNW1, HSNW2, and HSNW3 PCs. (**f**) Time-dependent PEC-WS of the HSNW2 PC for 12 h at a potential of −1 V versus RHE under illumination condition.

Figure 3a shows the open-circuit potential (OCP) of the photoelectrodes fabricated with the Ref-NW and the InGaN/GaN HSNWs as a function of time. The OCP of the photoelectrode fabricated with the Ref-NW decreases immediately after light illumination. This result indicates that the Ref-NW photoelectrode dominantly works as the photoanode (PA) [33]. On the other hand, the OCPs of the photoelectrodes based on the HSNWs samples increase immediately after illumination, indicating that they work as a PC without using p-type doping or a noble metal [33,34]. The changes in the OCP of the HSNW1, HSNW2, and HSNW3 PCs were measured as 7.9, 60.2, and 16.1 mV versus RHE, respectively. The high transition rate in the OCP curves indicates the efficient carrier extraction from the HSNW PCs to the electrolyte for the HER [35]. The rise (decay) times of the HSNW1, HSNW2, and HSNW3 PCs were calculated as 1.96 (2.01), 1.69 (1.86), and 2.52 s (2.82 s), respectively. The response times are defined as the times required for the potential to increase (decrease) from 10 (90) to 90% (10%) measured under illumination (dark) condition [20]. The response times are relatively short compared to those of the previous works [33,36]. The fast response characteristics of the PCs in this work indicate the high charge-transfer rate to the electrolyte [37]. Figure 3b,c show *J*–*V* characteristic curves of the Ref-NW PA and the HSNW PCs, measured under dark and illumination condition, respectively. The voltage was varied from −1 to 1 V versus RHE. The current densities at the potential of −1 V versus RHE for the Ref-NW PA and the HSNW1, HSNW2, and HSNW3 PCs were measured as 0.7, 1.6, 12.6, and 3.7 mA/cm^2^, respectively, under illumination condition. The current density was calculated by subtracting the dark current from the light current. The current densities of the InGaN/GaN-HSNW PCs are much higher than that of the Ref-NW PA, mainly due to the directional movement of the photo-generated carriers toward the electrolyte. Among the HSNW PCs, the HSNW2 PC shows the best PEC-WS performance compared to the HSNW1 and HSNW3 PCs, which is largely attributed to the crystal quality discussed in Figure 2e,f. Considering previous results summarized in Table 1, the InGaN/GaN HSNWs provide relatively high current density compared to those of previous approaches without using p-type doping or any metal catalyst [6,15,38,39,40,41,42,43,44]. Figure 3d shows the applied-bias photon-to-current conversion efficiencies (ABPEs) of the Ref-NW PA and the HSNW PCs, derived from the *J*–*V* characteristic curves. At the negative current-density region, the maximum ABPE values for the Ref-NW PA and the HSNW1, HSNW2, and HSNW3 PCs were calculated as 0.3 (−0.7 V), 0.4 (−1 V), 3.3 (−0.9 V), and 1.2% (−0.7 V), respectively. The ABPE value of the HSNW2 PC is higher than those of the others, which is also attributed to the crystal quality of the InGaN/GaN HSNWs. Moreover, the ABPE value is higher than those of the previous reports listed in Table 1 [38,39]. From the current densities and the ABPE values of the PA and PCs, the InGaN/GaN HSNW structure designed in this work to increase directional movement of photo-generated carriers toward the electrolyte can be an effective approach to improve the PEC-WS performance. Since the performance of PEC-WS is largely related to the degree of light absorption of photoelectrode media, the absorbance of the Ref-NW PA and HSNW PCs was measured and is shown in Figure 3e. The absorbance of the Si substrate without NW structures is also considered to investigate its effect on the PEC-WS. The light absorption at the Si substrate is negligibly small compared to those of the HSNW samples. This result is related that the absorption coefficient of Si is negligibly small compared to that of InGaN [45,46]. Considering the absorbance results, the effect of the Si substrate on PEC-WS is considered to be negligible. For the Ref-NW PA, the main absorption peak was observed at the wavelength of 350.7 nm. For the HSNW PCs, the absorption range is broadened in the visible region as the In content increases. However, the degree of the light absorption of the HSNW2 PC in the visible region is much higher than that of other samples, with the result that more electrons are generated and consequently accumulated in the InGaN-NW region. This increase in the number of effective carriers increases the reaction probability with the electrolyte and consequently improves the PEC-WS characteristics. Figure 3f shows the time-dependent properties of the HSNW2 PC, evaluated using chronoamperometry at the voltage of −1 V versus RHE. After 12 h of PEC-WS reaction, it was found that the current density of the HSNW2 PC was rarely decreased, which is largely attributed to the stable reaction of the InGaN/GaN HSNWs without significant structural degradation within the harsh electrolyte. The amount of hydrogen gas was calculated from the current density using the following Equation (1) [47]:

(1) $$\mathrm{Hydrogen}\;\mathrm{gas}\;\mathrm{production}=\frac{\frac{J\times t}{e}/2}{N_{A}}$$

where *J* is the measured current density (A/cm^2^), *t* is the measurement time (s), *e* is the carrier charge (C), and *N_A_* is the Avogadro constant (mol^−1^). The amount of hydrogen gas generated by the HSNW2 PC reached a maximum of 2.7 mmol/cm^2^ after operating the PEC-WS reaction for 12 h, which is much higher than those of the previous results summarized in Table 1 [15,38,40,41,44]. From these results it should be noted that InGaN/GaN HSNWs act as a highly efficient PC material. The structural properties including morphologies, lengths, and widths of the HSNWs significantly influence the PEC-WS performance, which will be discussed in more detail elsewhere.

**Figure 4:**
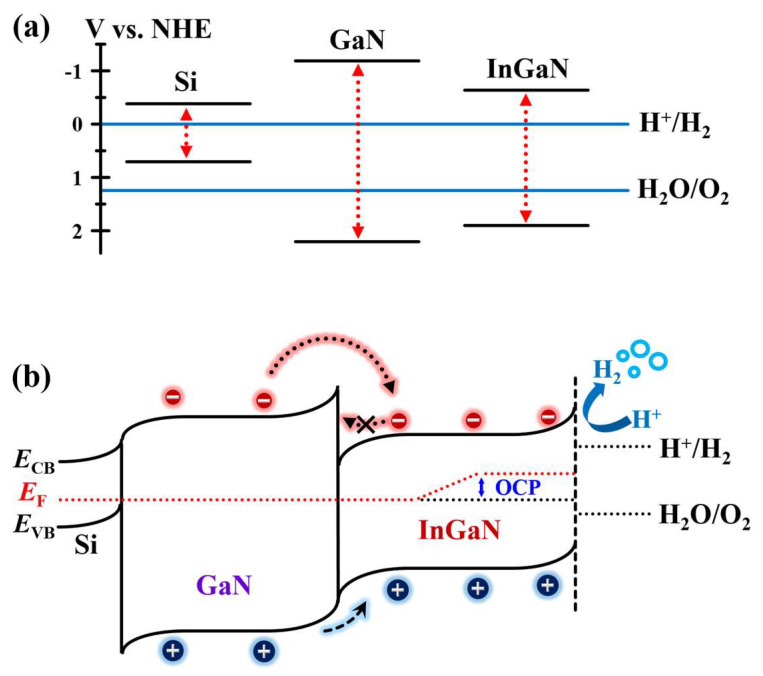
(**a**) Offset parameters with respect to NHE and (**b**) the energy-band structure of an InGaN/GaN HSNW for explaining the spatial behavior of photo-generated carriers at the hetero-interface between InGaN and GaN.

The high-performance PEC-WS using InGaN/GaN HSNW PC can be explained by the increase in the reaction probability of carriers with the electrolyte, largely due to the energy band alignment between InGaN and GaN. Figure 4a,b shows the offset parameters with respect to normal hydrogen electrode (NHE) and the theoretical energy-band structure of an InGaN/GaN HSNW grown on the Si(111) substrate, respectively. Theoretically, the difference between Fermi levels at the hetero-interface causes Fermi-level pinning [48], resulting that the energy-band structure is modulated as shown in Figure 4b. For the hetero-interface between GaN and Si, the type-II band alignment is formed and consequently promotes the separation of photo-generated carriers. On the other hand, at the hetero-interface between InGaN and GaN, the type-I band alignment is formed, resulting in the formation of an interfacial potential barrier [49]. Considering the interfacial potential barrier, the photo-generated electrons are accumulated in the InGaN-NW region and can move toward the electrolyte more easily. In addition, because the InGaN-NW region is spatially separated from the interface between Si and GaN NW in which defects are typically and largely formed [32], more photo-generated electrons can contribute to the PEC-WS reaction with the electrolyte. The carrier behavior in the InGaN/GaN HSNWs suggested in this work is quite different from that of the conventional InGaN/GaN HSNWs with a multiple quantum-well structure (MQW-NWs), in which InGaN is periodically positioned inside a GaN NW [50,51]. Even though there were reports on the use of InGaN/GaN MQW-NWs as a photoelectrode materials [50], it is suitable for the active medium of light-emitting devices because of their strong confinement of carriers [52]. That is, for the conventional MQW-NW photoelectrode, the reaction probability of carriers with the electrolyte may be limited to get high-performance PEC-WS. From this consideration, we adopted the InGaN/GaN HSNWs as a PC material using the directional migration of carriers and consequently increasing the PEC-WS performance.

**Figure 5:**
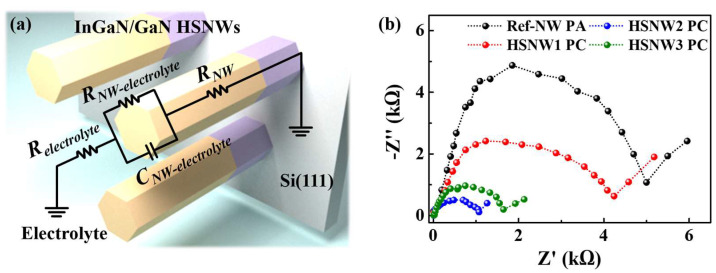
(**a**) Schematic and simplified equivalent circuit of an InGaN/GaN-HSNW PC. (**b**) Nyquist plots of the Ref-NW PA and HSNW1, HSNW2, and HSNW3 PCs measured a potential of −1 V versus RHE under illumination condition.

The high-performance PEC-WS of InGaN/GaN-HSNW PC is significantly influenced by the height of the potential barrier defined at the hetero-interface between InGaN and GaN along with the In content. To investigate reaction characteristics of the InGaN/GaN- HSNW PCs with respect to the In content, we approximated the interface between the InGaN/GaN HSNW and the electrolyte by an equivalent circuit with resistances and a capacitance. Figure 5a shows a schematic and a simplified equivalent circuit. In the equivalent circuit, a resistance and a capacitance, defined as the component that impedes the movement of electrons and the component that accumulates due to a change in the medium where electrons move, respectively, were quantitatively considered. The resistances for the electrolyte, the interface between the InGaN/GaN HSNW and the electrolyte, and the InGaN/GaN HSNW were denoted as *R_electrolyte_*, *R_NW-electrolyte_*, and *R_NW_*, respectively. The capacitance defined at the interface between the HSNW and the electrolyte was denoted as *C_NW-electrolyte_*. The interfacial resistance was quantified by analyzing the Nyquist plots. Figure 5b shows the Nyquist plots of the Ref-NW PA and the HSNW PCs measured at a potential of −1 V versus RHE in the frequency range from 100 kHz to 0.1 Hz under illumination condition. The InGaN/GaN-HSNW structure suggested in this study used to improve the reaction probability between electrons and electrolytes by controlling migration kinetics of carriers. Therefore, we mainly consider the interface between the InGaN region and the electrolyte for the InGaN/GaN-HSNW PC, rather than other interfaces. In the Nyquist plots the minor distortion from the ideal hemispherical shape is observed, which is attributed to the redox transition between the NWs and the electrolyte [53]. The *R_NW-electrolyte_* values, defined as the radius of semicircles in the Nyquist plots [54], for the Ref-NW PA and the HSNW1, HSNW2, and HSNW3 PCs were 5.1, 4.2, 1.0, and 1.6 kΩ, respectively. The *C_NW-electrolyte_* values for the Ref-NW PA and the HSNW1, HSNW2, and HSNW3 PCs, calculated from the imaginary component (−Z), were measured as 4.8, 2.5, 0.5, and 1.1 kΩ, respectively. The rate constant of interfacial charge transfer is defined as the reciprocal of the product of the resistance and the capacitance [55]. The HSNW2 PC showed the lowest interfacial resistance and capacitance among the NW samples, resulting that the interfacial charge-transfer rate is highest. This result indicates that the HSNW2 sample functions as the most effective PC material, which is also in good agreement with the *J*–*V* characteristic curves shown in Figure 3c.

## 4. Conclusions

We discussed the improvement in the PEC-WS performance by manipulating spatial migration direction of photo-generated carriers using high-crystalline InGaN/GaN HSNWs as a PC material. The PEC-WS using InGaN/GaN HSNWs showed the photocathodic properties with the current density of 12.6 mA/cm^2^ at −1 V versus RHE and the maximum ABPE value of 3.3% at −0.9 V versus RHE. These values are higher than those of previously reported NW-based PCs, indicating that highly efficient photocathodic properties were successfully obtained using simple InGaN/GaN HSNWs without p-type doping or any metal catalyst coating. The InGaN/GaN-HSNW PC increased the spatial accumulation of photo-generated carriers in the InGaN region due to the interfacial potential barrier formed at the hetero-interface between InGaN and GaN. As a result, the reaction probability with the electrolyte increases, resulting in the high-performance PEC-WS. Considering these results, the InGaN/GaN HSNW as a PC material would be an effective approach to achieve efficient PEC-WS technology.

## Figures and Tables

**Table 1:** Summary of the representative PEC-WS results of the previous PCs fabricated with various materials.

PC	Current Density (mA/cm^2^)	ABPE (%)	Hydrogen Evolution Rate (μmol/h·cm^2^)	Reference
p^+^-InGaN/TJ NW	~8 @ −0.3 V vs. RHE	-	~4	[15]
Cu_2_O NW	~10 @ −0.3 V vs. RHE	-	-	[6]
Si NW	~6 @ −1.2 V vs. RHE	1.14	~50	[38]
TiO_2_/InAs NW	~10 @ −1.2 V vs. RHE	1.9	-	[39]
CIS/AZO/TiO_2_	~0.7 @ 0 V vs. RHE	-	~175	[40]
Pt-CdS/CZTS	~6 @ −0.2 V vs. RHE	-	~55	[41]
Sb_2_Se_3_/TiO_2_/RuO_x_	~2 @ −0.2 V vs. RHE	3.6	-	[42]
Cu_2_O/Al_2_O_3_/TiO_2_/Pt	~7.8 @ 0 V vs. RHE	-	-	[43]
In_0.25_Ga_0.75_N NW	~5.3 @ −0.5 V vs. RHE	-	~50	[44]
InGaN/GaN HSNW	~12.6 @ −1 V vs. RHE	3.3	~225	This work
